# The effectiveness of the Guy’s Rapid Diagnostic Clinic (RDC) in detecting cancer and serious conditions in vague symptom patients

**DOI:** 10.1038/s41416-020-01207-7

**Published:** 2021-01-05

**Authors:** Saoirse Olivia Dolly, Geraint Jones, Paula Allchorne, Daniel Wheeler, Sunnyath Ali, Yaseen Mukadam, Sifan Zheng, Loay Rahman, Jan Sindhar, Charlotte Louise Moss, Danielle Harari, Mieke Van Hemelrijck, Anthony Cunliffe, Luigi Vincenzo De Michele

**Affiliations:** 1 Guy’s and St Thomas’ NHS Foundation Trust (GSTT), Medical Oncology, London, UK; 2 South East London Cancer Alliance, London, UK; 3 King’s College London, Faculty of Life Sciences and Medicine, London, UK; 4 King’s College London, School of Cancer and Pharmaceutical Sciences, Translational Oncology and Urology Research (TOUR), London, UK; 5 King’s College London, Geriatric and Acute Medicine, Department of Ageing and Health, London, UK; 6 Joint National Lead Macmillan GP Adviser and Joint Clinical Chair, South East London Cancer alliance, London, UK

**Keywords:** Cancer, Cancer

## Abstract

**Background:**

Rapid Diagnostic Clinics (RDC) are being expanded nationally by NHS England. Guy’s RDC established a pathway for GPs and internal referrals for patients with symptoms concerning for malignancy not suitable for a site-specific 2WW referral. However, little data assessing the effectiveness of RDC models are available in an English population.

**Methods:**

We evaluated all patients referred to Guy’s RDC between December 2016 and June 2019 (*n* = 1341) to assess the rate of cancer diagnoses, frequency of benign conditions and effectiveness of the service.

**Results:**

There were 96 new cancer diagnoses (7.2%): lung (16%), haematological (13%) and colorectal (12%)—with stage IV being most frequent (40%). Median time to definitive cancer diagnosis was 28 days (IQR 15–47) and treatment 56 days (IQR 32–84). In all, 75% were suitable for treatment: surgery (26%), systemic (24%) and radiotherapy (14%). Over 180 serious non-neoplastic conditions were diagnosed (35.8%) of patients with no significant findings in two-third of patients (57.0%).

**Conclusions:**

RDCs provide GPs with a streamlined pathway for patients with complex non-site-specific symptoms that can be challenging for primary care. The 7% rate of cancer diagnosis exceeds many 2WW pathways and a third of patients presented with significant non-cancer diagnoses, which justifies the need for rapid diagnostics. Rapid Diagnostic Centres (RDCs) are being rolled out nationally by NHS England and NHS Improvement as part of the NHS long-term plan. The aim is for a primary care referral pathway that streamlines diagnostics, patient journey, clinical outcomes and patient experience. This pilot study of 1341 patients provides an in-depth analysis of the largest single RDC in England. Cancer was diagnosed in 7% of patients and serious non-cancer conditions in 36%—justifying the RDC approach in vague symptom patients.

## Background

Five-year UK cancer survival rates are lower than comparable Western countries.^1–4^ This is multi-factorial, including cultural differences in health awareness, medical advice-seeking behaviours confounded by delays in diagnosis and treatment.^5^ Early identification of symptoms facilitating timely cancer diagnosis is linked with improved outcomes.^6–10^ The UK two-week wait (2WW) initiative aimed to reduce diagnostic and treatment intervals.^11^ This is based on recognition of red flag symptoms; however, only half of cancer patients ever develop these.^12^ Moreover, less than a quarter of cancer cases are diagnosed in this way,^13,14^ with a similar proportion presenting as emergencies^15,16^ and half in outpatient clinics.^17^ Many cancers present with vague or non-localising symptoms such as fatigue, weight loss or back pain,^18–22^ and are more likely to be diagnosed at a late cancer stage^12^ with higher mortality.^23^

**Fig. 1:**
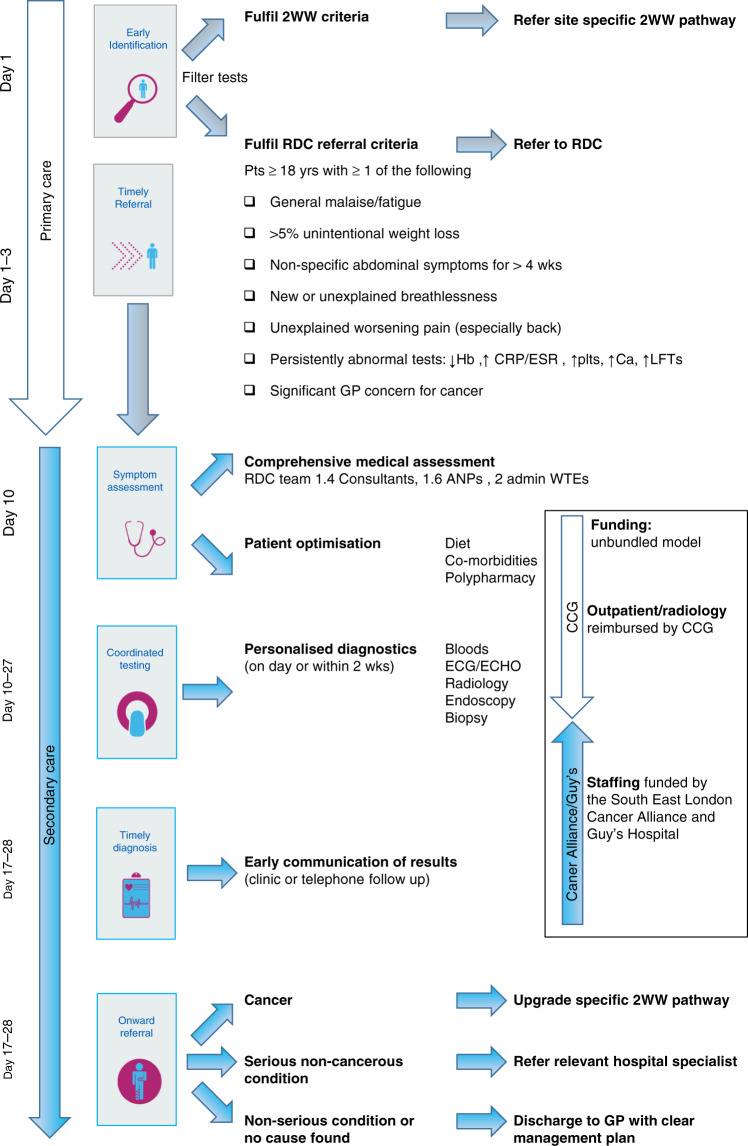
Timeline of clinical care runs vertically. Oversight of clinical responsibility for the patient is shown by the white arrow for primary care and blue arrow for secondary care. Funding is by the clinical commissioning group, cancer alliance and Guy’s hospital in the box. 2WW two week wait criteria, RDC rapid diagnostic clinic, ANP advanced nurse practitioner, ECG electrocardiogram, ECHO echocardiogram, CCG clinical commissioning group.

Rapid Diagnostic Clinics (RDCs) were based on the Danish three-legged cancer model.^24^ Patients with alarm symptoms were referred for fast-track assessment and those with a low risk of cancer referred back to the GP. The intermediate non-specific concerning group had a symptom screening questionnaire, upfront screening tests including blood, chest radiograph and abdominal ultrasound before being accepted into the RDC. Of this selected cohort, 16% of patients had new cancers diagnosed.^24,25^ In 2015, pilot UK Multi-Disciplinary Centres (MDCs) were established to improve cancer outcomes^26^ within the Accelerate Coordinate Evaluate (ACE) programme.^17,27–29^ The UK pilot was across 5 geographic locations, each with a distinct model, referral criteria and approach.^27,28^ Cancer detection rates were 4–16%, with serious benign conditions identified in a third.^17^ Concurrently, the Guy’s RDC was commissioned to establish a pathway for GPs and internal referrals for patients with symptoms concerning for malignancy not suitable for a site-specific 2WW referral. The collective aim was to deliver a personalised,^30^ co-ordinated diagnostic approach for adults with non-site-specific symptoms^31^ to facilitate earlier cancer diagnosis and fast-track care. The distinguishing feature of our RDC model, compared to ACE, is complete clinical oversight from an Internal Medicine Consultant. Patients undergo an upfront clinical review by a Consultant or Advanced Nurse Practitioner. This involves a comprehensive assessment of symptoms, co-morbidities, polypharmacy, risk factors, mental health as well as nutritional, functional and cognitive status. With this holistic approach, diagnostics are tailored to the individual and do not focus solely on cancer, as outlined in Fig. 1. This model is being rolled out nationally by NHS England and NHS Improvement, as part of the NHS long-term plan, with at least one Rapid Access Centre mandated per cancer alliance^32^ with full population coverage by 2024. The long-term plan to facilitate the faster diagnostic standard of 28 days,^33^ improved patient experience^34,35^ and reduced geographic variations in access to health services.

This retrospective audit evaluates the largest single -centre RDC in England with the primary aim to establish cancer detection rate. The secondary objectives included: (1) defining cancer type, stage and treatment rates, (2) determining if cancer patient characteristics or symptoms are unique compared to benign cases, (3) evaluating the rate and type of serious non-neoplastic conditions, (4) the clinical effectiveness of the RDC and (5) impact on diagnostic resources.

## Methods

### RDC model

Patients over 18 years of age are referred to Guy’s RDC if symptoms do not fit a specific 2WW criteria or fit multiple pathways (Fig. 1). Upfront assessment of symptoms, co-morbidities, polypharmacy, lifestyle factors and mental health is undertaken alongside optimisation of nutritional status and co-morbidities. Diagnostics are performed within 1–2 weeks to facilitate expedited diagnosis ideally within 1–3 weeks with prompt onward referral or discharge. See RDC pathway schematic in Fig. 1.

### Study population

This pilot study evaluates the Guy’s RDC model assessing complex symptom patients referred between December 2016 and June 2019 with data lock on 1st March 2020 (Fig. 1). Data were collected retrospectively from the electronic patient records (EPR) for all patients seen. Demographics, symptoms, laboratory test results, co-morbidities and lifestyle factors were collected, as well as cancer type, stage and treatment. The date of cancer diagnosis was the date of histological diagnosis or presumed radiological cancer diagnosis if biopsy was not undertaken. Serious non-cancerous conditions were defined as illnesses requiring onward secondary care referral. This study was approved by the Guy’s and St Thomas’ NHS Trust Quality Improvement and Clinical Audit Committee (Number 10236) and did not require regulatory ethics approval.

### Statistical methods

Descriptive statistics were employed to analyse the clinical characteristics of the cohort with Chi-squared and *t* tests. Variables assessed are outlined in Supplementary Table 1. The incidence of cancer, its type and frequency were calculated, and descriptive statistics used to analyse time to diagnosis and the number of investigations performed.

## Results

A total of 1341 prospective patients were seen in the Guy’s RDC between December 2016 and June 2019 (Table 1), 96% referred by GP and 4% internal referrals. Median follow-up was 18.69 months (13.21–26.55). Overall, patients had multiple co-morbidities (median 3, maximum 6), mental health issues (18%), polypharmacy (42%) and 20% were current smokers. They had multiple presenting symptoms (median 3, maximum 13) with weight loss (53.7%), pain (47.2%) and abdominal symptoms (36.1%) being most frequently reported, Table 1. The cancer patients (*n* = 93) were statistically significantly different to non-cancer patients (*n* = 1241) in terms of older age (mean 69 vs 62 years), being male (60.2% vs 39.3%) and with recent period of weight loss (1–4 months, 45% vs 25%). There is a high rate of social deprivation and current smoking (20–25%). Within the cancer group, there was a higher incidence of White ethnicity patients (56% vs 42%) and lower rate of Black ethnicity (7 vs 21%) in the cancer patients. There was also an increased rate of anaemia (43 vs 27%), thrombocytosis (18 vs 6%), raised CRP (68 vs 36%) and liver dysfunction (18 vs 8%) in cancer vs non-cancerous groups, Table 1.

**Table 1:** Patient characteristics.

	No cancer detected *N* = 1248	Cancer detected *N* = 93 (7.45%)	*p* value
*Age (mean S.D.)*	62.22 (15.42)	69.31 (11.21)	**<0.0001**
*Age categories (N(%))* < 60 years	568 (45.51)	23 (24.73)	**<0.0001**
≥60 years	680 (54.49)	70 (75.27)	
*Gender (N(%))* male	491 (39.34)	56 (60.22)	**<0.000**
Female	757 (60.66)	37 (39.78)	
*Death (N(%))* no/unknown	1235 (98.96)	69 (74.19)	0.42
Yes	13 (1.04)	24 (25.81)	
*Ethnicity (N(%))* White	519 (41.59)	52 (55.91)	**0.005**
Mixed	5 (0.40)	0 (0)	
Asian	71 (5.69)	4 (4.30)	
Black	253 (20.27)	6 (6.45)	
Other	18 (1.44)	3 (3.22)	
Missing	382 (30.61)	28 (30.11)	
*Co-morbidities (N(%)) n*one	155 (12.61)	10 (10.87)	0.19
1–2	393 (31.98)	28 (30.43)	
3–5	513 (41.74)	39 (42.39)	
>5	167 (13.59)	14 (15.22)	
Missing	1 (0.08)	1 (1.09)	
*BMI (mean S.D.)*	26.86 (19.23)	25.16 (6.24)	0.52
*Deprivation index (N(%))* 10% most deprived	11 (0.88)	0 (0)	0.39
10–20%	250 (20.03)	16 (17.20)	
20–30%	324 (25.96)	32 (34.41))	
30–40%	209 (16.75)	10 (10.75)	
40–50%	137 (10.98)	8 (8.60)	
50–60%	89 (7.13)	7 (7.53)	
60–70%	57 (4.57)	2 (2.15)	
70–80%	56 (4.49)	8 (8.60)	
80–90%	28 (2.24)	3 (3.23)	
10% Least deprived	20 (1.60)	2 (2.15)	
Missing	67 (5.37)	5 (5.38)	
*Polypharmacy (N(%))* no/unknown	721 (57.77)	54 (58.06)	0.96
Yes	527 (42.23)	39 (41.94)	
*Mental health illnesses (N(%))* no/unknown	1023 (81.97)	81 (87.10)	0.21
Yes	225 (18.03)	12 (12.90)	
*Smoking status (N(%))* non-smoker	697 (55.85)	43 (46.24)	0.06
Ex-smoker	138 (11.06)	17 (18.28)	
Current smoker	251 (20.11)	24 (25.81)	
Unknown	162 (12.98)	9 (9.68)	
*Alcohol intake (N(%))* within limits	261 (20.91)	19 (20.43)	0.80
Excessive (including prior excess)	126 (10.10)	12 (12.90)	
None	677 (54.25)	47 (50.54)	
Unknown	184 (14.74)	15 (16.13)	
*Number of symptoms (mean S.D.)*	2.94 (1.60)	3.16 (1.42)	0.19
*Symptom duration (N(%))* 2 weeks or less	12 (0.96)	1 (1.08)	**0.0002**
1 month or less	72 (5.77)	11 (11.83)	
1–3 months	235 (18.83)	31 (33.33)	
3–6 months	213 (17.07)	15 (16.13)	
6–12 months	170 (13.62)	14 (15.05)	
1 year +	150 (12.02)	12 (12.90)	
5 years +	14 (1.12)	0 (0)	
Missing	382 (30.61)	9 (9.68)	
*Fatigue (N(%))* none	916 (73.40)	69 (74.19)	0.93
G1	146 (11.70)	12 (12.90)	
G2	43 (3.45)	2 (2.15)	
G3	5 (0.40)	0 (0)	
Unspecified	138 (11.06)	10 (10.75)	
*Pain (N(%))* none	668 (53.53)	40 (43.01)	**0.0001**
G1	295 (23.64)	33 (35.48)	
G2	84 (6.73)	8 (8.60)	
G3	13 (1.04)	5 (5.38)	
Unspecified	188 (15.06)	7 (7.53)	
*Weight loss (N(%)) n*one	586 (46.96)	34 (36.56)	0.12
< 2 kg	24 (1.92)	4 (4.30)	
2 < 5 kg	84 (6.73)	11 (11.83)	
5 < 10 kg	143 (11.46)	10 (10.75)	
10 kg +	112 (8.97)	12 (12.90)	
Unspecified	299 (23.96)	22 (23.66)	
*Anaemia (N(%))* no/unknown	906 (72.60)	53 (56.99)	**0.001**
Yes	342 (27.40)	40 (43.01)	
*Haemoglobin (mean S.D.)*	109.28 (12.01)	108 (15.84)	0.60
*Thrombocytosis (N(%)) n*o/unknown	1172 (93.91)	76 (81.72)	**<0.0001**
Yes	76 (6.09)	17 (18.28)	
*Platelets (mean S.D.)*	501.55 (118.7)	537.47 (128.6)	0.045
*Raised inflammatory markers (N(%))* No/unknown	896 (71.79)	44 (47.31)	<0.0001
Yes	352 (28.21)	49 (52.69)	
*WCC (mean S.D.)*	11.54 (4.21)	10.07 (5.70)	0.57
*CRP (mean S.D.)*	25.73 (36.16)	55.50 (68.61)	<0.0001
*ESR (mean S.D.)*	53.94 (33.57)	56.00 (42.42)	0.86
*Hypercalcaemia (N(%))* no/unknown	1170 (93.75)	80 (86.02)	0.004
Yes	78 (6.25)	13 (13.98)	
*Calcium (mean S.D.)*	2.64 (0.11)	2.60 (0.09)	0.29
*Liver dysfunction (N(%))* no/unknown	1149 (92.07)	76 (81.72)	**0.0006**
Yes	99 (7.93)	17 (18.28)	
*Bilirubin (mean S.D.)*	37.76 (27.55)	74.25 (101.17)	0.18
*ALT/AST (mean S.D.)*	126.45 (112.63)	112.60 (98.08)	0.80
*ALP (mean S.D.)*	177.51 (55.13)	222.75 (82.21)	0.052

Statistically significant *p* values are highlighed in bold.

**Fig. 2:**
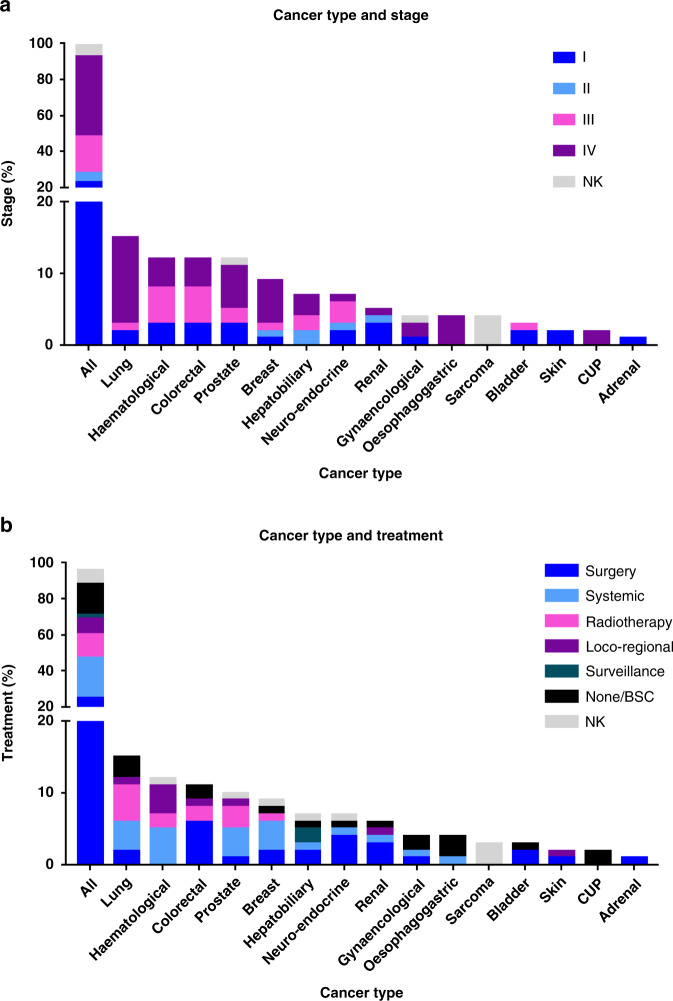
Cancers cases (*n* = 96) by stage and treatment. **a** Cancer type on x axis compared to percentage patients by tumour stage on the y axis. Stage I is dark blue, II light blue, III pink, IV purple and grey is not known (NK). **b** Cancer type on x axis compared to percentage of patients that received primary anti-cancer treatment. Treatments include surgery (dark blue), systemic anti-cancer treatment (light blue), radiotherapy (pink), loco-regional therapies such as ablation (purple), surveillance (dark green), none or best supportive care (BSC, black) and not known (NK, grey).

Ninety-six cases of cancer (7.3%) were detected with three patients having synchronous cancers (Fig. 2, Supplementary Table 2). The cancer incidence rate was 1.28 per 1000 person years. The commonest malignancies were lung (16.1%), haematological (12.9%) and colorectal (11.8%), Fig. 2a. Forty percent were metastatic presentations, whereas neuroendocrine, renal and bladder were mostly stage I (23–67%). Overall, 74% of patients were deemed fit to receive primary cancer treatment (Fig. 2b): surgery (26%), systemic anticancer treatment (24%) and radiotherapy (14%). Full cancer staging, histological subtype, primary treatment and performance status is outlined in supplementary table 2. Interestingly, of the newly detected cancers, only 8% fulfilled the tumour- specific 2WW referral criteria and a further 8% fulfilled a different pathway (Supplementary Table 2).

**Fig. 3:**
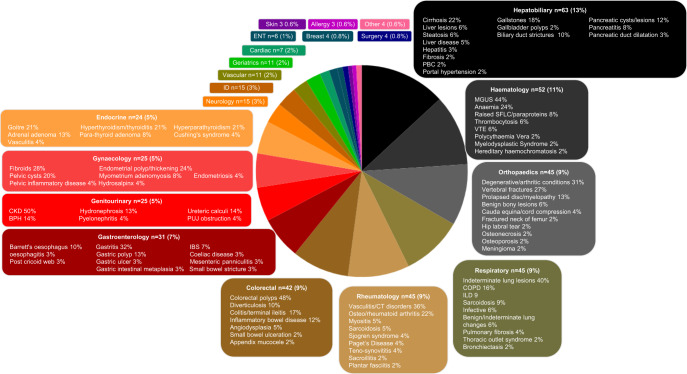
Serious benign conditions (*n* = 480); percentage (%) of patients affected by speciality. Pie chart highlighting the different specialities patients with non-cancer conditions were referred to which are colour coded with percentage volume of overall referalls. The more common specialities have a breakdown of the types of conditions diagnosed. The percentage in the box is by volume of that speciality. BPH benign prostatic hypertrophy, CKD chronic kidney disease, COPD chronic obstructive pulmonary disease, CT connective tissue disorders, ENT ear nose and throat, IBS irritable bowel syndrome, ID infectious diseases, ILD interstitial lung disease, PBC primary biliary cirrhosis, PUJ pelviureteric junction, MGUS monoclonal gammopathy of undetermined significance, SFLC serum free light chain, VTE venous thromboembolic disease.

Serious non-neoplastic conditions, defined by onward referral to a secondary care specialist, occurred in over a third (*n* = 480, 35.8%), Fig. 3. Over 180 conditions were diagnosed, and most frequent referring specialities were gastroenterology and colorectal (29%) and haematology (11%). Severe infectious diseases in 9 patients, including TB, HIV, Lyme disease and syphilis. Vascular conditions such as aortic aneurysms and dissection in 11 patients and vasculitis and connective tissue disorders in 16. Pre-malignant conditions in 6% included monoclonal gammopathy of undetermined significance (MGUS), colonic polyps and lung or liver nodules. Mild conditions or no organic cause was found in 57% (*n* = 768).

**Fig. 4:**
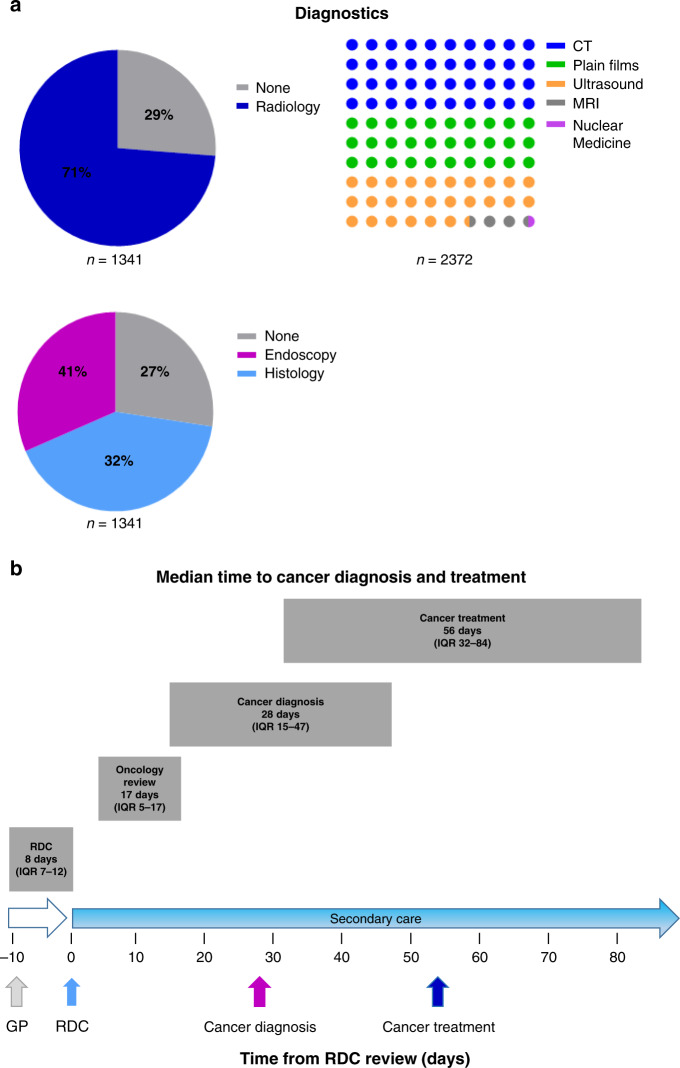
Rapid diagnostic clinic diagnostic resource use by a radiological and endoscopy with b median timescales to cancer diagnosis and treatment from RDC review. **a** It demonstrates resources used by the clinic. The two-colour pie chart shows the percentage of patients who underwent radiological investigations (blue) arranged by the RDC compared to those where no further tests were done (grey). The dot chart shows the proportion of radiological examinations undertaken. This includes CT (blue), plain radiographs (green), ultrasound (yellow), MRI (grey) and nuclear medicine tests (pink). The three-colour pie chart shows the percentage of patients who had endoscopy (pink), histological samples taken (blue) or none (grey). **b** The timeline from GP referral to RDC review and median cancer diagnosis and treatments. The grey boxes outlined the median value and the interquartile range (IQR).

In addition to the chest radiograph and optional abdominal ultrasound done on referral, three quarters of patients had radiological investigations arranged by the RDC amounting to 2,372 scans (Fig. 4a). The median number was 2 (IQR 1–3) with the most common investigations being CT (71%, Fig. 4b). Endoscopy and biopsies were undertaken in 41% and 32% of patients, respectively (Fig. 4c). Median time from referral to RDC appointment was 8 days (IQR 7–12 days). Median time to histological cancer diagnosis was 28 days and 56 days to primary treatment from RDC appointment (Fig. 4d).

## Discussion

This study evaluated the Guy’s RDC in 1341 consecutive vague symptom patients from an urban London population with in-depth analysis of cancer and non-cancerous conditions, and included 18.7 months median follow-up. This patient group is complex with multiple co-morbidities, high levels of polypharmacy, significant cancer risk factors, mental health conditions, ethnic variation and social deprivation. Cancer was detected in 7% with 31% being early stage (I–II) and 40% receiving radical treatment. Patients generally had similar characteristics, except the cancer patients were older, more likely to be male and have shorter symptom duration. Moreover, increased rates of smoking (25 vs 20%), anaemia (42 vs 27%) and thrombocytosis (18 vs 6%), compared to non-cancer controls. Over 180 serious non-neoplastic conditions were diagnosed in a third of patients necessitating referral to over 25 specialist teams. In total, 57% of patients had no significant cause identified and were discharged to their GP.

Compared to the Danish and ACE programmes, the Guy’s patients were younger with a median age of 62 (vs 69 and 65 years).^17,25^ Almost half presented after 3 months of symptoms, similar to ACE at 56% but much less than the Danish report of 13%.^25^ This suggests differences in medical seeking behaviours as well as primary care recognition and onward referral between countries. Our patient group had higher levels of deprivation and double the rate of smoking compared to the national average.^36^ There is no comparative data for the Central London RDC as the ACE annual reports were combined analyses of all RDCs.

The RDC cancer detection rate was 7%, higher than many 2WW pathways, including the Guy’s colorectal pathway of 3% (data not shown). In comparison to the colorectal cohort, the RDC patients were older (median 69 vs 61 years) with more patients from Black ethnic backgrounds (19% vs 15%). The Guy’s cancer conversion rate is similar to the Welsh 11%^37^ and ACE reports of 4–11%^27,28^ but less than the Danish model 16%.^25^ This likely reflects the high level of selection of patients into the Danish clinic. In contrast to the Guy’s open-referral criteria, all patients had to have persistent symptoms, complete pre-clinic questionnaire and have had upfront radiology before being accepted to the Danish clinic.^24,25^ The Welsh analysis was a smaller cohort of 186 patients, but all these patients underwent CT imaging^37^ compared to only 71% in our cohort. We feel medical assessment is pertinent to judge the risk of unnecessary radiation exposure against clinical concern, especially given the high proportion of patients with mild or non-organic causes.

Given that more than 95% of patients were treated within our network, we can report comprehensive data on cancer patients compared to more higher-level data in previous studies.^17,25^ The frequency of cancers detected correlates with the commonest malignancies; two-thirds were metastatic, which likely reflects later presentations in complex patients with vague symptoms. We also found less upper gastrointestinal cancers compared to ACE,^17^ which is likely due to differences in the referral criteria. Ours was broader than some more gastroenterology-specific MDCs. Additionally, we had a high rate of early urological cancers, amenable to curative resection, that were symptomatic, so no incidental radiological findings.

An NHS cancer priority is to improve early-stage cancer detection rates, and therefore increase radical treatment options.^6,10^ Similar to ACE, a third of patients in our RDC were detected at stage I–II with radical treatments in 40% primary surgery and systemic therapies. Moreover, pre-malignant conditions were diagnosed in 6%, including MGUS, indeterminate liver and lung lesions and gastrointestinal polyps. Although these are incidental findings, the prompt detection allows active monitoring and early treatment.

This study explicitly defined the serious non-cancer conditions not clearly outlined previously. More than 180 conditions were diagnosed, demonstrating the complexity of many diagnoses, as well as differing diagnostic labels hindering data collection. It also highlights the importance of the RDC in early diagnosis of chronic conditions and the importance of close links with multiple specialities to enable prompt specialist intervention.

Two-thirds of patients had no significant cause of their symptoms found (*n* = 768, 57%), either no organic cause identified or non-serious conditions that could be managed in primary care. This group was discharged to GPs with recommendations to review risks factors, polypharmacy and psycho-social circumstances. A fifth of patients had documented mental health conditions on referral. Clinical practice shows that this is underestimated in our population. Therefore, a collaboration is underway with the Integrating Mental & Physical health care: research, training & services (IMPARTS project) to assess the interplay of psychological and physical health.^38^

The Guy’s RDC is an effective service with a median time to histological cancer diagnosis of 28 days, in line with FDS standards. This is particularly pertinent for patients with vague symptoms who typically wait 34 days more for a diagnosis than those with red flag symptoms.^6^ These gains are due to more appropriate triage testing and a generalist rather than tumour group-specific approach, adequate diagnostic resources and efficient referral systems. Time to cancer diagnosis is longer than the ACE report.^17^ However, they defined it as the date of clinical diagnosis, which they reported in 217 of the 239 cancer patients diagnosed. Our definition was the date of histological confirmation. Radiological diagnosis was only used if a biopsy was deemed inappropriate to be undertaken. Median time to first cancer treatment was 28 days, which is due to RDC patients being upgraded to the site-specific 2WW pathway following the cancer diagnosis. We have since streamlined the service by implementing a system to track cancer patients to expedite specialist review. We are unaware of correlative published data for the vague symptom cancer cohort. Overall, ACE annual reports state a median of 57 days from referral to any treatment, whether cancer or benign, in a selected group of 142 of 2961 patients.^17^ Without peer-reviewed granular data, it is difficult to compare our outcomes. We calculated time to first treatment only for cancer patients using 2-week rule standards. We did not calculate this for the non-cancerous patients as it is difficult to define what constitutes treatment as it can vary from simple medications through to surgical interventions.

There are no randomised studies to show the effectiveness of RDCs, given the difficulty of having an appropriate control group. This large-scale English prospective study fully characterises non-specific symptom patients and their outcomes. We present comprehensive data on cancer and non-cancerous conditions. This study confirms better cancer detection rates comparable to straight-to-test models^17,24,25,27,28^ and exceeds many 2WW pathways.^39,40^ Only 16% of new cancers fulfil a 2WW referral criteria. This highlights the difficulty for GPs to identify the appropriate referral route and the importance of a vague symptom pathway. We have not been able to demonstrate the numbers of missed or subsequent cancer diagnoses in the RDC, but plan to assess this through the cancer registry.

This descriptive study outlines the demand on diagnostic services. It highlights the high volume of radiological and endoscopic investigations undertaken and the need for fast turnaround times to support the co-ordinated RDC approach. These descriptive statistics should help guide resource allocations in the RDS. Moreover, we were unable to estimate the impact of onward referrals for almost 600 patients to hospital specialists in terms of subsequent investigations and appointments, particularly within the overstretched NHS. We plan to assess this in future studies; however, we hypothesise that early definitive diagnosis within the RDC could potentially reduce primary and secondary care attendances.

Finally, it is important to note that the RDC holistic service will be paramount in the cancer recovery phase from the COVID-19 pandemic. Delays in patients presenting for medical advice alongside severely reduced access to diagnostics and cancer treatment will lead to a plethora of undiagnosed cancer cases.^41,42^ These will likely result in later-stage presentations with more complex symptomatology that may not fit a site-specific 2WW referral criteria. The RDC is well established to play a pivotal function in assisting expedited new cancer diagnoses, as well as support overwhelmed 2WW pathways to ensure that they can prioritise investigation of the high-risk patients clinically fit for investigation and subsequent oncological treatment.

In conclusion, these data confirm that RDCs provide a streamlined pathway for complex vague symptom patients. In total, 7% were diagnosed with cancer and 36% with serious non-cancerous conditions that can be challenging to diagnose in primary care. The commonest cancers found were lung, haematological and colorectal with a third at an early stage (I–II) and 40% were amenable to radical treatment. Median times to cancer diagnosis and treatment were 28 and 56 days, respectively, in line with current NHS targets. A prospective evaluation of the RDC with health economic studies is needed to evaluate the effectiveness with the RDS national expansion by NHS England and Improvement. Moreover, RDCs could be pivotal in the cancer recovery phase of the COVID-19 pandemic.

## Supplementary information

Supplementary Files

Infographic of figure 3.

## Data Availability

Anonymised data may be available from the corresponding author on request.
