# Supplementary material for: The effectiveness of the Guy’s Rapid Diagnostic Clinic (RDC) in detecting cancer and serious conditions in vague symptom patients
Source: Br J Cancer. 2021 Jan 5;124(6):1079–87. doi: 10.1038/s41416-020-01207-7 (PMC7783491; doi:10.1038/s41416-020-01207-7)
Supplement: Supplementary file 1 — Supplementary Files [file 41416_2020_1207_MOESM1_ESM.docx]

| **Main exposure variable** | **Minimal adjustments** |
| --- | --- |
| Age | - Continuous - < 60 and ≥ years |
| Ethnicity | - White, Black, Asian, Mixed, Other, Missing |
| Deprivation Index | - Increments of 10% |
| Polypharmacy (> 5 medications) | - No/unknown, yes |
| History of mental health illness | - No/unknown, yes |
| Smoking | - Non, current, ex, unknown |
| Alcohol intake | - Within limits, excessive, non, unknown |
| Number of presenting symptoms | - 2 weeks or less, 1 month or less, 1-3 months, 3-6 months, 6-12 months, 1 year +, 5 years +, Missing |
| Fatigue | - None, G1, G2, G3, unspecified |
| Pain | - None, G1, G2, G3, unspecified |
| Weight loss | - None, <2kg, 2<5kg, 5<10kg, >10kg, unspecified |
| Anaemia | - No/unknown, yes - Value |
| Thrombocytosis | - No/unknown, yes - Value |
| Raised inflammatory markers – CRP, ESR | - No/unknown, yes - Value |
| Hypercalcaemia | - No/unknown, yes - Value |
| Liver dysfunction – Bili, AST/ALP, ALP | - No/unknown, yes - Value |

**Supplementary Table 1:** Overview of demographic and clinical characteristics dataset of Rapid Diagnostic Clinic patients

| Type | Subtype  n (%) | Histology  n (%) | Cancer Stage  n (%) | | | | | WHO performance status  n (%) | | | | | Primary treatment modality  n | | | | | | | Fulfils 2WW  criteria |
| --- | --- | --- | --- | --- | --- | --- | --- | --- | --- | --- | --- | --- | --- | --- | --- | --- | --- | --- | --- | --- |
|  |  |  | I | II | III | IV | NK | 0 | 1 | 2 | 3 | NK | Systemic | RT | Surgery | Local | W&W | NK | BSC or None | Site specific  (other site) |
| All cancer cases  n 96 (7.2%)^#^ |  |  | 22  (23) | 8  (8) | 17  (18) | 39  (40) | 11  (11) | 5  (5) | 35  (38) | 14  (15) | 16  (17) | 23  (25) | 22 | 13 | 25 | 2 | 9 | 8 | 17 | 8  (8) |
| Lung  15 (16.1%) | NSCLC 13 (87)  SCLC 1 (7)  Mesothelioma 1(7)~ | Adenoca 10 (67)  Squamous 2 (13)  Carcinoma 1 (7)  Small Cell 1 (7)  Epithelioid mesothelioma 1 (7) | 2  (13) | 0  (0) | 1  (7) | 12  (80) | 0  (0) | 0  (0) | 2  (14) | 4  (27) | 4  (27) | 3  (20) | 4 | 5 | 2 | 0 | 1 | 0 | 3 | 1 |
| Haematological 12 (12.9%) | HL 2 (17)  DLBCL 1 (8)  Follicular 3 (25)  CLL/SLL 1 (8)  Lymphoplasmacytic 1 (8)  Myeloma 4 (33) | | 2  0  1  -  -  0 | 0  0  0  -  -  2 | 0  0  2  -  -  1 | 0  0  0  -  -  0 | 0  1  0  -  -  1 | 2 | 2 | 2 | 0 | 5 | 5 | 5 | 2 | 0 | 0 | 4 | 1 | 4  (1) |
| Colorectal 11 (11.8%) | Colon 7 (47)^+^  Rectum 3 (20)^+^  Anal 1 (7) | Adenoca 9~ (82)  Squamous 1 (9)  NK 1 (9) | 3  (20) | 0  (0) | 5  (33) | 4  (27) | 0  (0) | 0  (0) | 3  (20) | 3  (20) | 2  (13) | 0  (0) | 0 | 2 | 6 | 0 | 1 | 0 | 2 | 2 |
| Prostate 10 (10.8%) | Gl 1 2 (18), Gl 2 1 (9), Gl 3 1 (9)  Gl 4 0 (0), Gl 5 4 (36), NK 3 (27) | | 3  (27) | 0  (0) | 2  (18) | 6  (55) | 1  (9) | 0  (0) | 1  (5) | 0  (0) | 0  (0) | 5  (33) | 4 | 4 | 3 | 1 | 0 | 1 | 1 | 0 |
| Breast  9 (9.7%) | IDC 5 (56), ILC 1 (11) ^^^, Adenoca 1 (11)  Carcinoma 1 (11), NK 1 (11) | | 1  (11) | 1  (11) | 1  (11) | 6  (67) | 0  (0) | 2  (13) | 5  (33) | 0  (0) | 1  (7) | 1  (7) | 4 | 4 | 1 | 2 | 0 | 0 | 1 | 0 |
| HPB 7  (7.5%) | CCA 3 (43)  Pancreas 1 (14)  HCC 3 (43) | Adenoca 1  Adenosq 1 Carcinoma 1  Adenoca 1 | 0  (0) | 2  (29) | 2  (29) | 3  (43) | 0  (0) | 0  (0) | 2  (29) | 2  (29) | 3  (43) | 0  (0) | 1 | 0 | 2 | 2 | 0 | 1 | 1 | 0  (1) |
| Neuro-endocrine 7 (7.5%) | Lung 1 (14)  Small bowel 3 (43)  Pancreatic 2 (29)  Prostate 1 (14) | G1 4 (57)  G2 2 (29)  G3 0 (0)  NK 1 (14) | 2  (29) | 1 (14) | 3  (43) | 1  (14) | 0  (0) | 1  (14) | 4  (57) | 1  (14) | 0  (0) | 0  (0) | 1 | 0 | 4 | 0 | 0 | 1 | 1 | NA  (2) |
| Renal 6  (6.5%) | Clear cell 5 (83) ^~^  Angiomyolipoma 1 (17) | | 3  (60) | 1  (20) | 0  (0) | 1  (20) | 0  (0) | 0  (0) | 5  (100) | 0  (0) | 0  (0) | 0  (0) | 1 | 1 | 0 | 3 | 0 | 1 | 0 | NA  (3) |
| Gynae 4  (4.3%) | Cervical 1 (25)  Endometrial 1 (25)  Ovarian 2 (50) | NK  G1 endometrioid  HG ovarian 2 | 1  (25) | 0  (0) | 0  (0) | 2  (50) | 1  (25) | 0  (0) | 2  (50) | 0  (0) | 2  (50) | 0  (0) | 1 | 0 | 1 | 0 | 0 | 0 | 2 | 0 |
| OG 3  (3.2%) | Oeso 1 (33) ^^^  Gastric 2 (67) | G3 3 (100) | 0  (0) | 0  (0) | 0  (0) | 4  (100) | 0  (0) | 0  (0) | 1  (33) | 0  (0) | 2  (67) | 0  (0) | 1 | 0 | 0 | 0 | 0 | 0 | 3 | 0 |
| Sarcoma 3 (3.2%) | Retroperitoneal 1 (33) - NK  Bone Spindle cell 1 (33)  Abdominal de-differentiated liposarcoma 1 (33) | | - | - | - | - | - | 0  (0) | 1  (33) | 0  (0) | 1  (33) | 1  (33) | 0 | 0 | 0 | 0 | 0 | 0 | 3 | NA |
| Bladder 3 (3.2%) | TCC 1 (33)  Papillary 1 (33)  NK 1 (33) | G1 0 (0)  G2 1 (33)  G3 1 (33)  NK 1 (33) | 2  (67) | 0  (0) | 1  (33) | 0  (0) | 0  (0) | 0  (0) | 1  (33) | 0  (0) | 1  (33) | 1  (33) | 0 | 0 | 2 | 0 | 0 | 0 | 1 | 1 |
| Skin 2 (2.2%) |  | BCC 2 | 2 | 0 | 0 | 0 | 0 | 1 | 1 | 0 | 0 | 0 | 0 | 0 | 1 | 0 | 1 | 0 | 0 | 0 |
| CUP 2 (2.2%) |  | Adenoca 1  NK 1 | 0 | 0 | 0 | 2 | 0 | 0 | 0 | 0 | 2 | 0 | 0 | 0 | 0 | 0 | 0 | 0 | 2 | NA  (1) |
| Adrenal 1 (1.1%) |  | Phaeochromocytoma 1 | 1 | 0 | 0 | 0 | 0 | 0 | 0 | 0 | 0 | 1 | 0 | 0 | 1 | 0 | 0 | 0 | 0 | NA |

Supplementary Table 2: New cancer diagnoses by tumour type, stage and treatment modality

* Cancer staging (TNM classification version 7), except International Staging System for myeloma.

^#^ Three patients had synchronous cancers ^~^mesothelioma and renal; ^+^colon and rectal; ^^^oesophageal and breast

W&W watch and wait, CUP carcinoma of unknown primary, NSCLC non small cell lung cancer, SCLC small cell lung cancer, G grade, IDC invasive ductal carcinoma, ILC invasive lobular carcinoma, Adenoca adenocarcinoma, NK not known, HL Hodgkin’s lymphoma, DLBCL Diffuse large B cell lymphoma, CLL/SLL Chronic lymphocytic lymphoma/chronic lymphocytic leukaemia, CCA cholangiocarcinoma, HCC hepatocellular carcinoma, oeso oesophageal, TCC transitional cell carcinoma, 2WW two week rule referral
