# Supplementary material for: The effectiveness of the Guy’s Rapid Diagnostic Clinic (RDC) in detecting cancer and serious conditions in vague symptom patients
Source: Br J Cancer. 2021 Jan 5;124(6):1079–87. doi: 10.1038/s41416-020-01207-7 (PMC7783491; doi:10.1038/s41416-020-01207-7)
Supplement: Supplementary file 2 — Infographic of figure 3. [file 41416_2020_1207_MOESM2_ESM.pptx]

## Slide 1
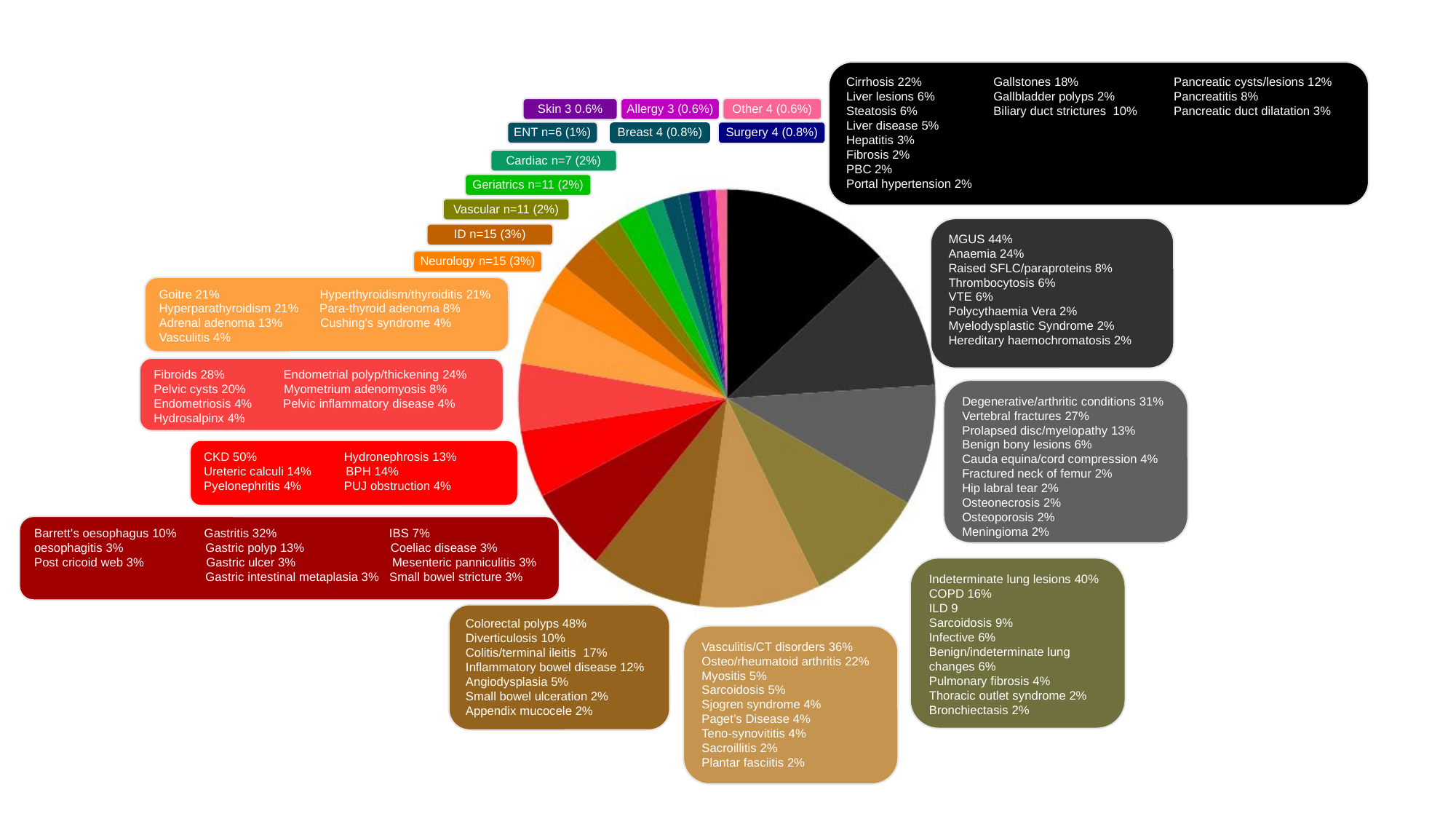

Cirrhosis 22%	 Gallstones 18%	Pancreatic cysts/lesions 12%
Liver lesions 6%	 Gallbladder polyps 2%	Pancreatitis 8%
Steatosis 6%	 Biliary duct strictures 10%	Pancreatic duct dilatation 3%
Liver disease 5%
Hepatitis 3%
Fibrosis 2%
PBC 2%
Portal hypertension 2%
Skin 3 0.6%
Allergy 3 (0.6%)
Other 4 (0.6%)
ENT n=6 (1%)
Surgery 4 (0.8%)
Breast 4 (0.8%)
Hepatobiliary
n=63 (13%)
Haematology
n=52 (11%)
Endocrine
n=24 (5%)
Gynaecology
n=25 (5%)
Orthopaedics
n=45 (9%)
Genitourinary
n=25 (5%)
Gastroenterology
n=31 (7%)
Respiratory
n=45 (9%)
Colorectal
n=42 (9%)
Rheumatology
n=45 (9%)
Neurology n=15 (3%)
Cardiac n=7 (2%)
Rheumatology
9%
Geriatrics n=11 (2%)
Rheumatology
9%
Vascular n=11 (2%)
Rheumatology
9%
MGUS 44%
Anaemia 24%
Raised SFLC/paraproteins 8%
Thrombocytosis 6%
VTE 6%
Polycythaemia Vera 2%
Myelodysplastic Syndrome 2%
Hereditary haemochromatosis 2%
ID n=15 (3%)
Rheumatology
9%
Goitre 21% 	 Hyperthyroidism/thyroiditis 21%
Hyperparathyroidism 21% Para-thyroid adenoma 8%
Adrenal adenoma 13% Cushing's syndrome 4%
Vasculitis 4%
Fibroids 28% Endometrial polyp/thickening 24%
Pelvic cysts 20% Myometrium adenomyosis 8%
Endometriosis 4% Pelvic inflammatory disease 4%
Hydrosalpinx 4%
Degenerative/arthritic conditions 31%
Vertebral fractures 27%
Prolapsed disc/myelopathy 13%
Benign bony lesions 6%
Cauda equina/cord compression 4%
Fractured neck of femur 2%
Hip labral tear 2%
Osteonecrosis 2%
Osteoporosis 2%
Meningioma 2%
CKD 50% 	 Hydronephrosis 13%
Ureteric calculi 14% BPH 14%
Pyelonephritis 4%	 PUJ obstruction 4%
Barrett's oesophagus 10% Gastritis 32%	 IBS 7%
oesophagitis 3%	 Gastric polyp 13% Coeliac disease 3%
Post cricoid web 3% Gastric ulcer 3% Mesenteric panniculitis 3%	 Gastric intestinal metaplasia 3% Small bowel stricture 3%
Indeterminate lung lesions 40%
COPD 16%
ILD 9
Sarcoidosis 9%
Infective 6%
Benign/indeterminate lung changes 6%
Pulmonary fibrosis 4%
Thoracic outlet syndrome 2%
Bronchiectasis 2%
Colorectal polyps 48%
Diverticulosis 10%
Colitis/terminal ileitis 17%
Inflammatory bowel disease 12%
Angiodysplasia 5%
Small bowel ulceration 2%
Appendix mucocele 2%
Vasculitis/CT disorders 36%
Osteo/rheumatoid arthritis 22%
Myositis 5%
Sarcoidosis 5%
Sjogren syndrome 4%
Paget’s Disease 4%
Teno-synovititis 4%
Sacroillitis 2%
Plantar fasciitis 2%
